# Multi-wave-mixing-induced nonlinear modulation of diffraction peaks in an opto-atomic grating

**DOI:** 10.1038/s41598-020-73825-3

**Published:** 2020-10-08

**Authors:** Bibhas Kumar Dutta, Pradipta Panchadhyayee, Indranil Bayal, Prasanta Kumar Mahapatra, Nityananda Das

**Affiliations:** 1 Department of Physics, Sree Chaitanya College (WB State University), North 24 Parganas, Habra, WB 743 268 India; 2 Department of Physics (UG & PG), Prabhat Kumar College (Vidyasagar University), Contai, Purba Medinipur 721404 India; 3 ITER, Siksha ‘O’ Anusandhan University, Bhubaneswar, Odisha 751030 India; 4 Department of Physics, J. K. College (Sidho Kanho Birsha University), Purulia, WB 723 101 India

**Keywords:** Optical physics, Quantum optics

## Abstract

We propose an atomic model in close-loop configuration, which exhibits controllable *symmetric* and *asymmetric* evolution of significantly enhanced diffraction peaks of the weak probe beam in an opto-atomic grating at far-field regime. Such results are obtained by the linear and nonlinear modulation of the intensities of the diffraction peaks as a result of multi-wave-mixing-induced modification of spatially modulated coherence in a closed four-level atomic system. Novelty of the results lies in predicting the diffraction pattern with uniform peak height due to the dominance of the amplitude part of the grating-transfer-function at the condition of exact atom-field resonance, which is unique to the present model. Efficacy of the present scheme is to apply it in producing nonlinear light generated by four-wave-mixing-induced control of spatially modulated coherence effect. The work also finds its importance for its applicability in the field of all-optical devices.

## Introduction

With the advancement of laser based science and technology, it has been possible to construct new optical device like Electromagnetically Induced Grating (EIG)^1–3^. In the case of Electromagnetically Induced Diffraction (EID), any aperture, or obstacle responsible for diffracting light beam can be built directly or indirectly by one or more external electromagnetic fields^1^. The formation of a grating as a result of the way of applying the external fields interacting resonantly with an atomic medium gives rise to the concept of electromagnetically induced grating (EIG). In contrast to the four-wave-mixing (FWM) induced grating^1–3^, the features of spatially modulated absorption and transparency of a laser beam passing through a coherently prepared atomic medium are explored to demonstrate EIG^4–8^ both theoretically and experimentally at far-field regime, where $z>>D^{2}/{\lambda }$; *z* being the distance of probing the output between the grating and the image plane, *D*, the lateral dimension of the aperture and $\lambda$, the wavelength of the incident wave.

The phenomena like Electromagnetically Induced Transparency (EIT)^9^, double-EIT (DEIT)^10^, Electromagnetically Induced Absorption (EIA)^11^, laser-controlled Decay Interference Induced Coherence (DIIC)^12^ may lead to obtain *spatially* *modulated* *atomic* *coherence* under the *standing*-*wave* *field* configuration. Owing to such coherence effects, a number of works^4–8,13–26^ have presented EIG in various atomic models. More specifically, EIG in terms of spatially modulated EIT or DEIT has been analyzed in Refs.^4–8,13,14,18,20,22–25^ and EIG based on EIA has been explained in Ref.^5,17^. Impact of the DIIC effect on the spatially modulated coherence is reported in Refs.^15,16,19^ and the gain-assisted control of EIG has been shown in Ref.^21^. Contribution of coherent phase-modulation of the transfer function, which is attributed to the presence of standing-wave regime, has been shown to be essential for the enhancement of the first and second order peaks as compared to the central peak^4,7,8,15–20^. The appearance of EIG with Rydberg atoms has also been discussed in Refs.^23,24^. It is worth mentioning that, in case of Rydberg atoms, one can obtain the phenomenon of EIG for the excited states wherein strong optical nonlinear effect is induced due to dipole-dipole interaction. In the present model our objective is to generate nonlinear generation of diffraction peaks as a result of the non-degenerate four-wave mixing (FWM) process where the excited energy levels are different from those used in Rydberg atoms. In recent days, photonic graphene lattice originated via the effect of EIT in an atomic vapor cell is a highly promising candidate for generation of various nonlinear optical effects^27–29^. Evolution of optical $\mathcal{{PT}}$ -symmetry has been employed to construct EIG in Raman-Nath regime as investigated in Ref.^30,31^. Cross-grating like structures based on two-dimensional (2D) EIG is described in Refs.^32–34^. Experimental observation of the diffraction pattern in a 2D-optically induced atomic lattice is reported^35^ in a three-level atomic system. The mechanism of electromagnetically induced Talbot effect similar to EIG has been discussed in Ref.^36^ and also demonstrated experimentally^37^.

Following the generic models in rarefied medium a good number of attempts have been made to explore the phenomenon of EIG in solid state media such as, semiconductor quantum nanostructures^38–44^. A different method of obtaining dual EIG has been studied on the basis of spatially modulated biexciton coherence in Ref.^40^. Phase-dependent generation of EIG has been addressed also in those works^40,41^. A new scheme based on semiconductor quantum dot and metal nanoparticle hybrid system is proposed to realize EIG^42,43^. In a tripod-type atomic model the appearance of dual EIG is also reported in the presence of an incoherent pump field^45^.

**Figure 1:**
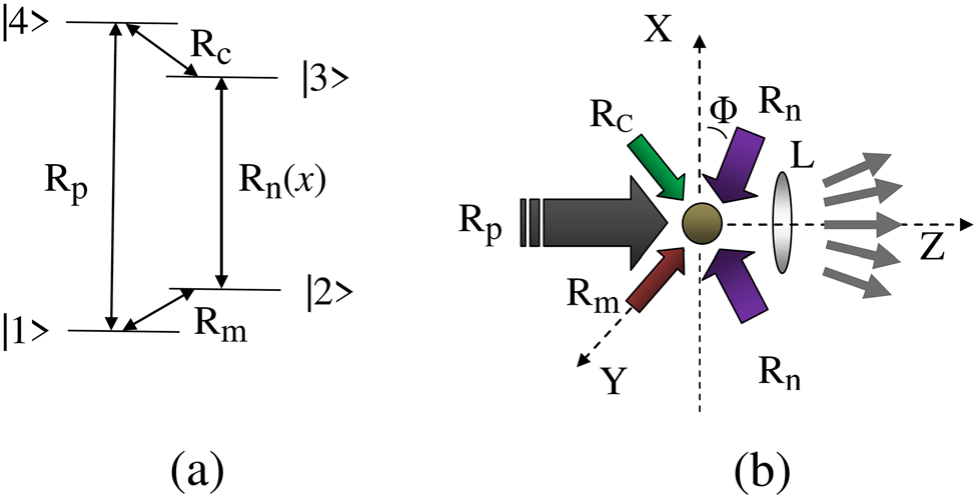
(**a**) Field-coupled energy level diagram of a four-level atomic system. $R_j$ ($j = p, m, n, c$) denotes the Rabi frequency for the applied fields. Specifically, $R_n(x)$ denotes the position dependence of the Rabi frequency (see text). (**b**) Schematic view of possible field arrangement with the atom (circular spot) placed at the centre. Angle $\phi$ denotes the orientation of the components of the control fields forming standing wave. *L* indicates a lens. Diverging arrows are shown for different orders of diffraction.

The mechanism of EID rendering EIG as presented in this article may lead to construct a device called opto-atomic grating for the study of diffraction of laser at high frequency region. The scheme of EIG can be chosen to be an useful technique to generate nonlinear light almost free from background-noise as reported in Ref.^2,3^ for detection of light signal based on the FWM technique. To this aim, electromagnetically induced far-field diffraction of a weak coherent optical beam is investigated after passing it through an atomic system interacting with the assembly of travelling and standing wave fields as shown in Fig. 1a,b. Fig. 1a represents the field-coupled energy level diagram for a four-level close-loop interaction scheme. In various parameter conditions the linear response of a weak probe field is modified by nonlinear effects generated as a consequence of *multi*-*wave*-*mixing*-*induced* *coherence* (MWMIC). It is worth pointing that, in absence of the field applied between the levels $|1>$ and $|2>$, the given model appears like a DEIT model. But as soon as this field is switched on, FWM-induced coherence will emerge as a result of the four-level loop linkage in the present model leading to non-degenerate FWM generation in a close-loop configuration. In this article we have shown that the generation of MWMIC significantly controls the diffraction of the weak probe beam through the opto-atomic slits originated by spatially modulated coherence in the standing-wave regime.

In the proposed scheme, generation of spatially modulated coherence is regulated not only by the DEIT, but by the MWMIC also. Such controlling process, at the condition of exact atom-field resonance, is shown to be unique to the given four-level model in inducing a uniform distribution of multiple peaks in the diffraction pattern where peaks of all the orders have almost equal magnitude with significant sharpness. The amplitude part of the grating-transfer-function plays the vital role in forming such peak pattern. It has been shown that a small shift from the condition of exact resonance results in the nonlinear peak modulation due to the effect of MWMIC and in turn controls the asymmetry in the alignment of the higher order peaks. Contextually, we note that the diffraction pattern of $\mathcal{{PT}}$-symmetry induced grating is observed to be asymmetric. In contrast to the basic mechanism of $\mathcal{{PT}}$-symmetric grating, we have shown the asymmetric evolution of diffraction peaks in the presence of MWMIC effect. We have examined the explicit role of the Rabi frequencies and detuning on the intensities of the higher order peaks. The present model can be used as an effective technique for generation and detection of nonlinear light on the basis of FWM-induced control of spatially modulated coherence effect. Overall, the diffraction patterns as obtained in this work seem to be plausible in practice for all-optical devices and concur to the specific demand of optically-induced atomic lithography.

## Theoretical model

The field-coupled energy-level configuration of the atom is shown in Fig. 1a, where the transitions $|1>$-$|2>$, $|1>$-$|4>$, $|2>$-$|3>$ and $|3>$-$|4>$ are influenced by the field induced Rabi frequencies $R_m=\frac{{{\bar{\mu }}}_{21}.{\bar{\epsilon }}_m}{2\hbar }$, $R_p=\frac{{{\bar{\mu }}}_{41}.{\bar{\epsilon }}_p}{2\hbar }$, $R_n(x)=R_nsin({\pi }px/{{\Lambda }_c})$ with $R_n=\frac{{{\bar{\mu }}}_{32}.{\bar{\epsilon }}_n}{2\hbar }$, and $R_c=\frac{{{\bar{\mu }}}_{43}.{\bar{\epsilon }}_c}{2\hbar }$, respectively. Here, $\mu _{jk}$ denotes the dipole moment associated with the corresponding transition, while ${\epsilon _j}/2$ for the field amplitude. The field probing the coherence induced by the control fields in the system is designated by the Rabi frequency $R_p$. Spatial dependence of the Rabi frequency denoted as $R_n(x)$ originates from the standing wave formed by the counter-propagating components of the coupling field (Fig. 1b) defined as $E_n(x,t)=\frac{\bar{\epsilon }_n}{2}sin(k_c x cos\phi )$ $e^{i\omega _nt}$ + $c.c.=$ $\frac{\bar{\epsilon }_n}{2}sin({\pi }px/{{\Lambda }_c})$ $e^{i\omega _nt}$ + *c*.*c*.; where $p=cos{\phi }$, and ${\Lambda }_c={{\lambda }_c}/2$ with ${\lambda }_c$ being the wavelength of the coupling field. We note that ${\Lambda }^{\prime }_c(={{\Lambda }_c}/p)$ implies the separation between two consecutive nodes, or antinodes. By changing the angle $\phi$ the value of $\Lambda ^{\prime }_c$ can be varied. Other control fields of Rabi frequencies $R_m$ and $R_c$ are treated to be the travelling waves like the probe field in the present model.

In view of finding the validity of the given model in a realistic atomic system, we have chosen the field-induced transitions^46^ for Rubidium (^87^Rb) $D_1$ lines ($5^2S_{1/2}$ $\leftrightarrow$ $5^2P_{1/2}$). The dipole-allowed transitions $|1>$-$|4>$ and $|2>$-$|3>$ coincide with $(S)F = 1$ $\leftrightarrow$ $(P)F = 2$ and $(S)F = 2$ $\leftrightarrow$ $(P)F = 1$ transitions. Dipole forbidden transitions $|1>$-$|2>$ and $|3>$-$|4>$ correspond to $(S)F = 1$ $\leftrightarrow$ $(S)F = 2$ and $(P)F = 1$ $\leftrightarrow$ $(P)F = 2$ are taken into account in the presence of microwave field. Note that ^87^Rb is selected as a suitable candidate for versatile applications in the field of quantum optics and laser spectroscopy. In experimentation with laser cooled atoms Rb has a vast applicability due to the presence of several ground states with long lifetimes, and easy accessibility of the lowest excited state with diode lasers.

The coherent part of the atom-field interaction is described by the Hamiltonian under the electric dipole and the rotating wave approximations as

1 $$\begin{aligned} {{\mathcal {H}}}(t)= & {} -{\hbar }[\Delta _m |2>
<2| + (\Delta _p - \Delta _c) |3>
<3| + \Delta _p |4>
<4| + (R_m |1>
<2| + R_p |1>
<4| \nonumber \\&+ R_n(x) |2>
<3| + R_c |3>
<4| + c.c)] \end{aligned}$$

where $\Delta _m=\Delta _p - \Delta _c - \Delta _n$ with the detuning parameters $\Delta _p=\omega _{p} - \omega _{41}$, $\Delta _c=\omega _{c} - \omega _{43}$, $\Delta _n=\omega _{n} - \omega _{32}$ and $\Delta _m=\omega _{m} - \omega _{21}$. The system dynamics can be explained by the semiclassical density matrix equation as given by

2 $$\begin{aligned} \frac{\partial {\rho }}{\partial t} = -\frac{i}{\hbar }[{{\mathcal {H}}},\rho ] + \Lambda \rho \end{aligned}$$

where the term $\Lambda \rho$^14^ includes the effect of incoherent decay-mechanism inherent to the present atomic model. The required off-diagonal density matrix equations are presented as follows

3 $$\begin{aligned} {\dot{\rho }}_{41}= & {} -Z_{41} {\rho }_{41} + iR^*_p (\rho _{11} - \rho _{44}) + iR^*_c \rho _{31} -iR^*_m \rho _{42} \end{aligned}$$

4 $$\begin{aligned} {\dot{\rho }}_{31}= & {} -Z_{31} {\rho }_{31} + iR^*_n(x) \rho _{21} + iR_c \rho _{41} - iR^*_m \rho _{32} - iR^*_p \rho _{34} \end{aligned}$$

5 $$\begin{aligned} {\dot{\rho }}_{21}= & {} -Z_{21} {\rho }_{21} + iR^*_m (\rho _{11} - \rho _{22}) + iR_n(x) \rho _{31} - iR^*_p \rho _{24} \end{aligned}$$

where $Z_{41}=\Gamma _4 - i\Delta _p$, $Z_{31}=\Gamma _3 - i(\Delta _p - \Delta _c)$ and $Z_{21}=\Gamma _2 - i(\Delta _p - \Delta _c - \Delta _n)$. Here, $\Gamma _4 = (\gamma _{41} + \gamma _{42})/2$, $\Gamma _3 = (\gamma _{31} + \gamma _{32})/2$ where $\gamma _{mn}$ ($m=3,4$ and $n=1,2$) denotes the natural decay rate. $\Gamma _2$ is considered to incorporate small coherence dephasing rate.

Under weak-field approximation, we treat the Rabi frequencies $R_p$ and $R_m$ to the first order and the others ($R_n$ and $R_c$) to all orders and the given set of density matrix equations can be solved in steady state to obtain the expression of ${\rho }^{(1)}_{41}$ on the basis of following conditions to be satisfied: $\rho ^{(0)}_{11}\approx 1$, $\rho ^{(0)}_{42}=$ $\rho ^{(0)}_{32}=\rho ^{(0)}_{34}=0$, and $\rho _{jk}=$ $\rho ^*_{kj}$. By making the substitutions: $\rho ^{(1)}_{41}=$ ${\tilde{\rho }}_{41}e^{-i\phi _p}$, $R_p=|R_p|e^{i\phi _p}$, $R_m=|R_m|e^{i\phi _m}$, $R_n(x)=|R_n(x)|e^{i\phi _n}$, $R_c=|R_c|e^{i\phi _c}$ with the condition $\phi _p=\phi _m + \phi _n + \phi _c$, we obtain

6 $$\begin{aligned} \frac{{\tilde{\rho }}_{41}}{|R_p|} = f_L + f_{NL1} + f_{NL2} \end{aligned}$$

with

$$\begin{aligned} f_L= & {} i\frac{Z_{21}Z_{31}}{Z_{21}Z_{31}Z_{41} + Z_{21}|R_c|^2 + Z_{41}|R_n(x)|^2},\\ f_{NL1}= & {} i\frac{|R_n(x)|^2}{Z_{21}Z_{31}Z_{41} + Z_{21}|R_c|^2 + Z_{41}|R_n(x)|^2},\\ f_{NL2}= & {} -i\frac{|R_n(x)||R_m||R_c|/|R_p|}{Z_{21}Z_{31}Z_{41} + Z_{21}|R_c|^2 + Z_{41}|R_n(x)|^2}, \end{aligned}$$

where $f_L$ indicates the linear response of the probe field, and the two nonlinear terms $f_{NL1}$ and $f_{NL2}$ are responsible for cross-phase modulation and inducing MWMIC in the probe response. The first nonlinear term $f_{NL1}$ contains two driving fields $R_c$ and $R_n(x)$, while the second nonlinear term $f_{NL2}$ holds another control field $R_m$ in addition to $R_c$ and $R_n(x)$. We note that a close-loop configuration cannot be set up without the application of $R_m$ between the levels $|1>$ and $|2>$. Being populated by all the field terms involved in our model the $f_{NL2}$ term bears the signature of four-level loop linkage. Thus, $f_{NL2}$ carries special significance regarding the origination of MWMIC and projects importance of choosing this specific four-level close-loop model from other usual four-level models like N-type, Y-type, inverted Y-type, and so on. Another aspect is to mention that this close-loop model must lead to the emergence of a collective phase. In an attempt to generate and optimise effective FWM-induced effect, the establishment of maximal coherence (i.e., optimal MWMIC) must be ensured. This is possible only when the collective phase will be zero.

The polarization induced in the probe transition is given by performing the quantum average over the corresponding transition moment^12,14^ as follows

7 $$\begin{aligned} {{\mathcal {P}}}_p = \epsilon _0 \chi _p \epsilon _p = 2N{\mu }_{14}{\tilde{\rho }}_{41} \end{aligned}$$

where $\epsilon _0$ being the free-space permittivity and *N*, the atomic density. The susceptibility $\chi _p$ is expressed as

8 $$\begin{aligned} \chi _p = \frac{N|{\mu }_{14}|^2}{\epsilon _0 \hbar \gamma _{41}} \chi \end{aligned}$$

with

9 $$\begin{aligned} \chi = \frac{\gamma _{41}{\tilde{\rho }}_{41}}{|R_p|} = \frac{\gamma _{41}}{|R_p|}\frac{(GU-HV)+i(GV+HU)}{U^2+V^2} \end{aligned}$$

where

$$\begin{aligned} G= & {} |R_p|[\Gamma _2(\Delta _p - \Delta _c) + \Gamma _3(\Delta _p - \Delta _c - \Delta _n)],\\ H= & {} |R_p|[\Gamma _2\Gamma _3 - (\Delta _p - \Delta _c)(\Delta _p - \Delta _c - \Delta _n) + |R_n(x)|^2] - |R_n(x)||R_m||R_c|],\\ U= & {} \Gamma _2\Gamma _3\Gamma _4 - \Gamma _2\Delta _p(\Delta _p - \Delta _c) - (\Delta _p - \Delta _c - \Delta _n)[\Gamma _3\Delta _p + \Gamma _4(\Delta _p - \Delta _c)] + \Gamma _2|R_c|^2 + \Gamma _4|R_n(x)|^2,\\ V= & {} \Gamma _4(\Gamma _3[\Delta _p - \Delta _c - \Delta _n) + \Gamma _2(\Delta _p - \Delta _c)] + \Delta _p[\Gamma _2\Gamma _3 - (\Delta _p - \Delta _c)(\Delta _p - \Delta _c - \Delta _n)] + (\Delta _p - \Delta _c - \Delta _n)|R_c|^2 + \Delta _p|R_n(x)|^2. \end{aligned}$$

In order to obtain the self-consistent equation for the probe field propagating through the atomic medium, we consider the probe field with a planar wavefront travelling along the *z*-direction, which is represented as $E_p=\frac{1}{2}\epsilon _pe^{i(\omega _pt - k_pz)}+c.c.$. The amplitude factor $\epsilon _p$ is assumed to remain unchanged in the transverse direction (*x*-direction) during the propagation of the wave though the atomic medium. We define the propagation vector, $k_p=\frac{2\pi }{\lambda _p}$; ${\lambda _p}$ being the wave length of the probe field. Now, the Maxwell’s equation for the probe field is given in the following form^12^

10 $$\begin{aligned} \frac{\partial {\epsilon _p}}{\partial {z}} = i\frac{\pi }{\epsilon _0 \lambda _p} {{\mathcal {P}}}_p = i C \frac{\chi }{\lambda _p} {\epsilon _p} \end{aligned}$$

where $C=\frac{{\pi }N|{\mu }_{14}|^2}{\epsilon _0{\hbar }{\gamma _{41}}}$ is defined as a dimensionless constant by following the Ref.^12,47^, which implies the optical depth of the probe field. For the sake of simplicity of the calculation, *C* is chosen to be unity. The value of C conforms to the values of the parameters: $|{\mu }_{14}| \approx 3\times 10^{-29}$ C-m, $\gamma _{41} \approx 6\times 10^6$ Hz and $N \approx 10^{12}$ atoms /cc. The product term $i\chi$ is defined as $A+i D$ where absorption $A=-\frac{\gamma _{41}}{R_p}\frac{(GV+HU)}{U^2+V^2}$, and dispersion $D=\frac{\gamma _{41}}{R_p}\frac{GU-HV}{U^2+V^2}$. We introduce the dimensionless distance ($\xi$) and relate it with the optical depth of probe field (*C*) through the expression, $\xi =z C/{\lambda _p}$. Eq. (10) can then be recast as

11 $$\begin{aligned} \frac{\partial {\epsilon _p}}{\partial {\xi }} = (A + i D) \epsilon _p, \end{aligned}$$

which leads us to obtain the transmission function as spatially-modulated-grating transfer function

12 $$\begin{aligned} T({\xi },x) = T_o e^{[A(x)+i D(x)] \xi } \end{aligned}$$

where $T_o$ is a constant. For convenience, we consider the normalized transfer function $f({\xi },x) = T({\xi },x)/T_o$, which plays the vital role in producing the grating spectrum. Because, all the information about coherent and incoherent atom-field interactions is stored in this function. This is to mention here that $|f({\xi },x)|$ *i*.*e*. $e^{A(x) \xi }$ is the amplitude part of the grating-transfer-function, while the term $\Phi ({\xi },x)$ ($=D(x)\xi$) evolves as the phase part of the grating-transfer-function^4^. If the value of the amplitude transfer function becomes predominant over the phase part of the transfer function i.e., *A* dominates over *D* in magnitude, we obtain the intensity distribution of the grating mostly induced by the absorption. In such a situation we obtain the amplitude grating or absorption grating. With the variation of the system-parameters, if the intensity distribution pattern be dramatically changed due to the significant contribution of the phase part of the transfer function, i.e., *D* dominates over *A* in magnitude, we obtain the intensity distribution of a phase grating.

We define the spatial width of the probe beam as the product-term $M\Lambda _c^{\prime }$. *M* is a non-zero positive integer, which implies the allowed number of opto-atomic slits. According to Fig. 1b, an opto-atomic slit assembly is formed in the *x*- direction. The nodal regions where probe suffers no interaction in the absence of spatially modulated field act as slits, whereas the antinodes can be treated as opaque regions due to the interaction of the probe in the presence of spatially modulated field. The amplitude of the probe field $\epsilon _p$ is taken to be uniform over the width of the beam. On transmission through the slit-assembly, the spatial modulation can be attributed to the probe amplitude by introducing the function $G(x)=\epsilon _pf({\xi },x)$. If $\theta$ be the angle of diffraction of the probe field from the *z*-direction, then the Fourier transform of *G*(*x*) gives rise to the resultant amplitude of the Fraunhofer diffraction pattern as given below^48^

13 $$\begin{aligned} A_P({\theta }) \propto \int _{-\infty }^{\infty } G(x)\,\, exp\left( -i\frac{2{\pi }}{\lambda _p} x sin{\theta }\right) dx \end{aligned}$$

Introducing the dimensionless space-variable $x^{\prime }=\frac{x}{{\Lambda }_c}$, we newly define the parameter $Q=\frac{{\Lambda }_{c}^{\prime }}{\Lambda _p}$, where $\Lambda _p={\lambda }_p/2$. Thus the intensity of the diffraction pattern is expressed after the algebraic simplification as follows^14,49^

14 $$\begin{aligned} I(\theta ) = I_o |F(\theta )|^2 \frac{\sin ^2(M{\pi }Q\sin \theta )}{M^2\sin ^2({\pi }Q\sin \theta )} \end{aligned}$$

where $I_o$ is the constant of proportionality including $|\epsilon _p|^2$ and $\Lambda _c^2$, and the function $F(\theta )$ is given by^48,50^

15 $$\begin{aligned} F(\theta ) = \int _{-{1/2}}^{1/2} f(L) exp(-ip{\pi }Qx^{\prime }\sin \theta ) dx^{\prime } \end{aligned}$$

where *p* is defined earlier. In accordance to Eq.(14) the position of the *n*-th order diffraction maxima corresponds to the grating equation, $Q\sin \theta =n$. The grating equation implies that the number of diffraction peaks within the whole range of $sin\theta$ equals $(2Q+1)$ with peak positions at $sin\theta = n/Q$; $-Q \leqslant n \leqslant Q$. The larger the value of *Q*, larger will be the number of diffraction peaks within the whole range of $sin\theta$. For higher value of *M*, the spatial width of the probe beam will be larger, which makes it possible to get large number of diffraction peaks depending upon the value of *Q*. Thus, both the parameters *M* and *Q* play the key role in controlling the number of diffraction peaks. The length *L* indicates the dimensionless distance traversed by the probe field through the active medium. We note that $|F(\theta )|^2$ implies the intensity distribution of the single slit diffraction. In practice, for carefully chosen atomic transition to be probed, $\Lambda ^{\prime }_c$ may be of the order of $\Lambda _p$, because one needs to adjust the value of *Fresnel* *number*^14^ to be much less than unity in the far-field diffraction regime.

## Results and discussions

**Figure 2:**
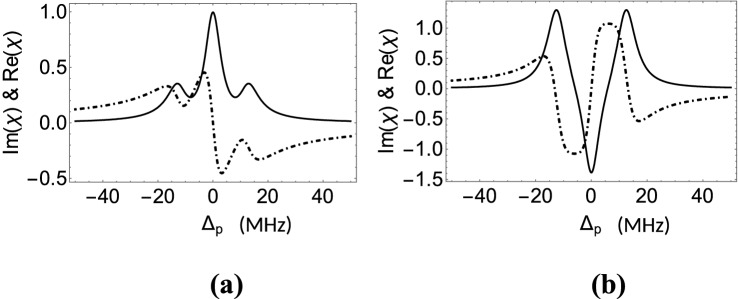
Variation of Real and Imaginary parts of $\chi$ with respect to the probe detuning ($\Delta _p$): (**a**) $R_m=0$, (**b**) $R_m=0.03$ MHz. Other parameters: $R_p=0.01$ MHz, $R_n=10$ MHz, $R_c=8$ MHz, and $\Delta _n=\Delta _c=0$. The solid and dashed dot lines indicate Im($\chi$) and Re($\chi$), respectively.

**Figure 3:**
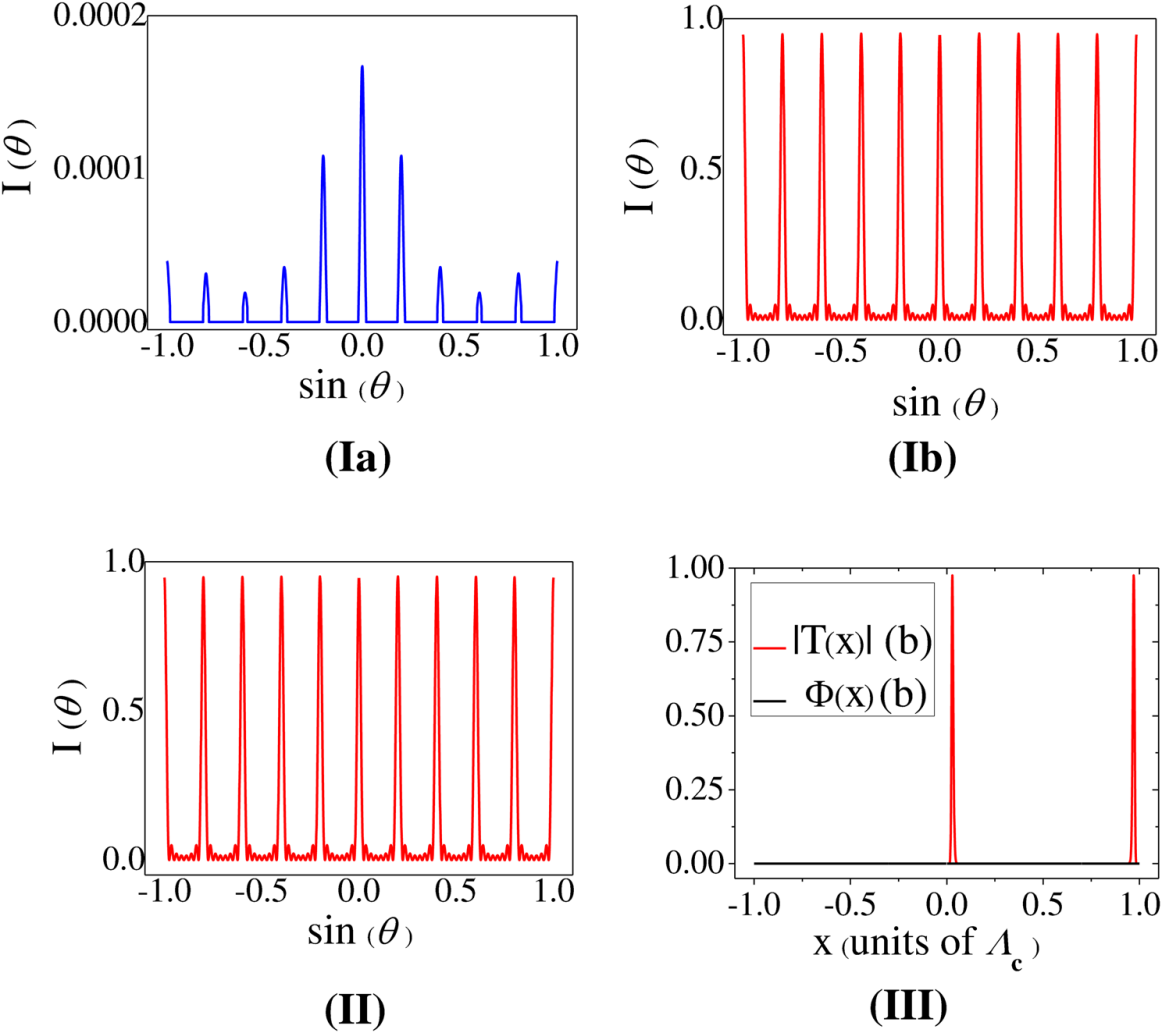
Resonant evolution of grating-spectra: I: Plot of Diffraction Intensity versus sin($\theta$)—(**a**) $R_m=0$, (**b**) $R_m=0.03$ MHz. Other parameters: $R_p=0.01$ MHz, $R_n=10$ MHz, $R_c=8$ MHz, $L=30$, $\Delta _p=\Delta _n=\Delta _c=0$, *Q* = 5, and *M* = 8. II: Plot of Diffraction Intensity versus sin($\theta$) with same conditions as in I(**b**) considering only the nonlinear contribution ($f_{NL2}$ in the expression of $\frac{\rho _{41}}{R_p}$ (Eq. 6)). III: Plot of Transfer Function *T*(*x*) (Red Colour) and phase $\Phi$ (Black Colour) with same parameter conditions as in I(**b**).

**Figure 4:**
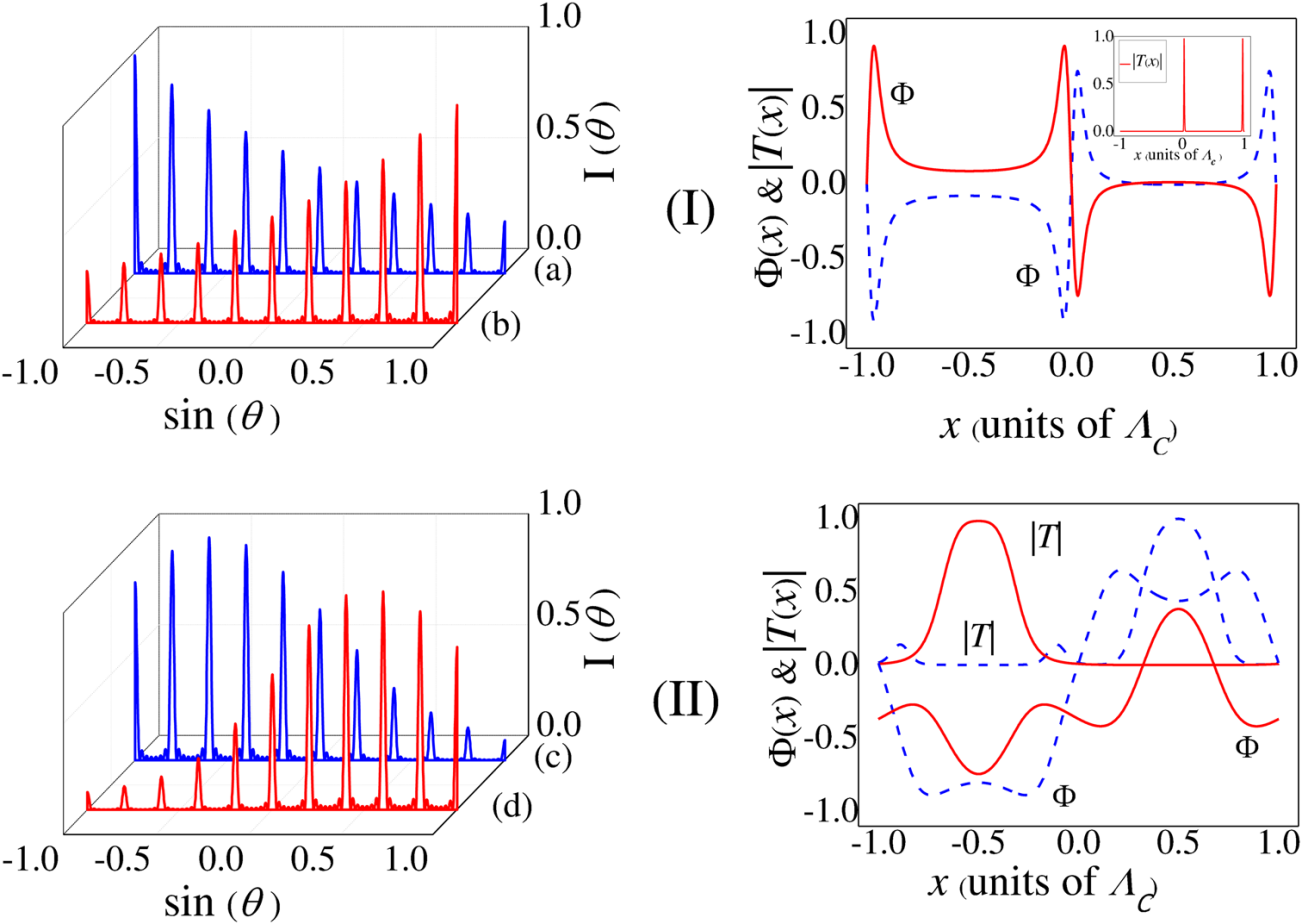
Near resonant evolution of grating-spectra: I: Left Panel—Plot of Diffraction Intensity versus sin($\theta$)—(**a**)$\Delta _p=0.1$ MHz, and (**b**)$\Delta _p=-0.1$ MHz; Right Panel: Corresponding plot of Transfer Function *T*(*x*) (Inset) and phase $\Phi$. In both the intensity $I(\theta )$ and phase ($\Phi$) plots blue-line and red-line graphs represent the parameter conditions (a) and (**b**), respectively whereas red-line graph (Inset) indicates *T*(*x*) profile (idential for (**a**,**b**)). II: Left Panel—Plot of Diffraction Intensity versus sin($\theta$)—(**c**) $\Delta _p=4$ MHz, and (**d**) $\Delta _p=12$ MHz; Right Panel: Corresponding plot of Transfer Function *T*(*x*) and phase $\Phi$. Blue-line and red-line graphs in (II) indicate the Intensity $I(\theta )$ plots in the right panel for the parameter conditions (**c**,**d**), respectively. Same colours are used in the left panel to indicate the corresponding *T*(*x*) and $\Phi$ plots. Other parameters: $R_p=0.01$ MHz, $R_m=0.03$ MHz, $R_n=10$ MHz, $R_c=8$ MHz, *Q* = 5, $M=8$, and $L=30$.

**Figure 5:**
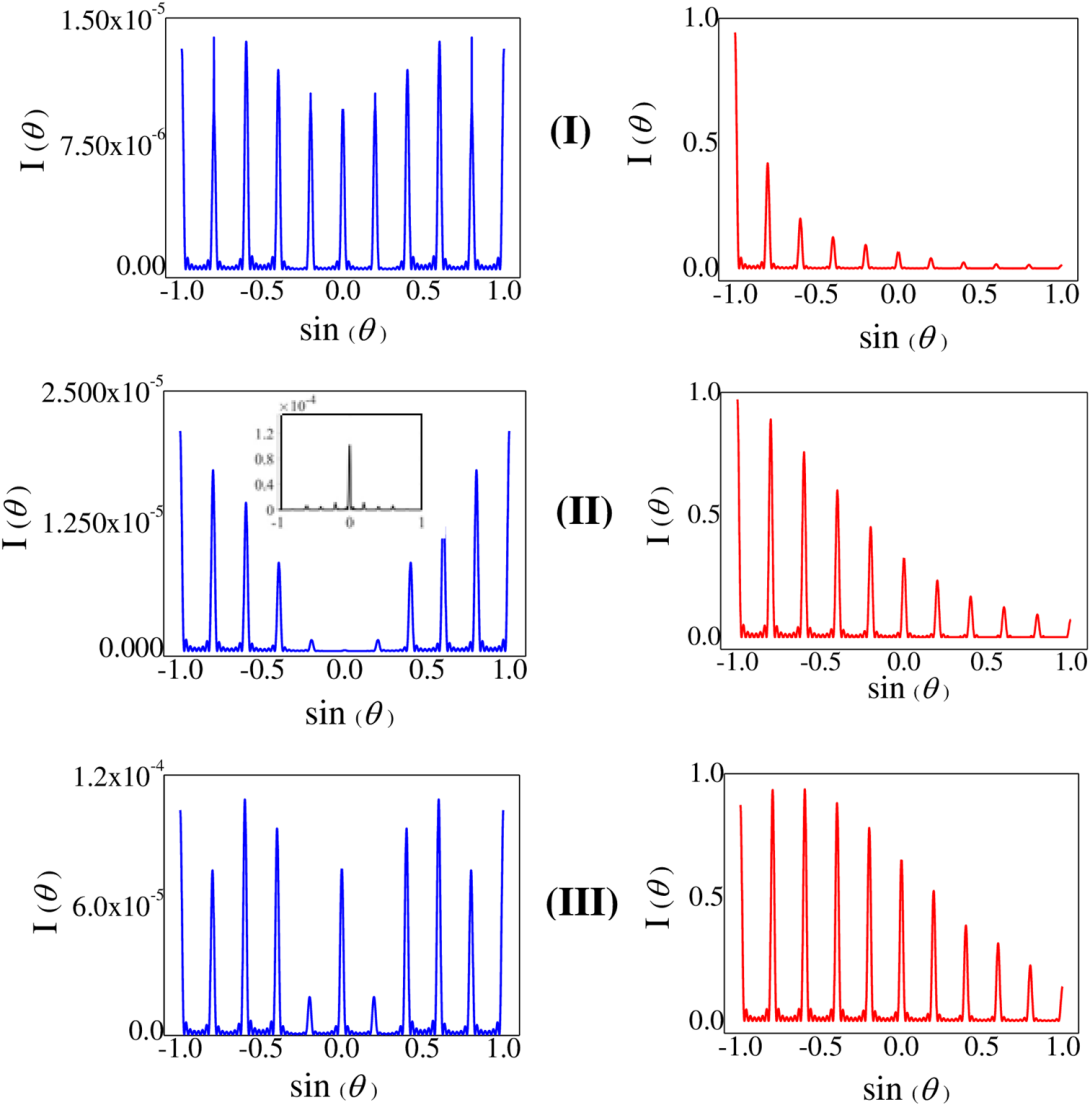
Sample length dependence of grating-spectra at near resonance: (I) $\Delta _p=1.5$ MHz and $L=60$, (II) $\Delta _p=3.5$ MHz and $L=70$, (III) $\Delta _p=5.2$ MHz and $L=70$, with the other parameters: $R_p=0.01$ MHz, $R_n=10$ MHz, $R_c=8$ MHz, $\Delta _n=\Delta _c=0$, *Q* = 5, and *M* = 8. All the blue-line graphs in the left panel are drawn for $R_m=0$ and the red-line graphs in the right panel for $R_m=0.02$ MHz.

We have computed numerically the Fraunhofer diffraction pattern of the probe beam by using Eqs. (6), (12), (14) and (15). First of all, we set the values of $Q=5$ and $M=8$. The value of *Q* indicates that the number of peaks including the central one is 11 within the whole range of $sin\theta$. The natural decay rates of the excited levels $|3>$ and $|4>$ are considered to be equal to 6 MHz. For all the results presented here we set the Rabi frequency of the probe laser as $R_p$ = 0.01 MHz and choose $\gamma _{31}=\gamma _{32}=\gamma _{42}=\gamma _{41}$ = 6 MHz and $\Gamma _2$ = 0.05 MHz. The values of the Rabi frequencies of the other two fields are taken as $R_n=10$ MHz and $R_c=8$ MHz in Figs. 2, 3, 4, 5. In the present work, one of our objectives is to find out the impact of MWMIC in the diffraction profile. The maximal value of MWMIC can be ensured through effective generation of FWM only when the two driving fields, $R_c$ and $R_n(x)$ will be resonantly tuned. That is why we consider the deuning condition $\Delta _c=\Delta _n=0$ throughout the work. In this condition the robustness of the field-atom interaction is also enhanced in the present model. The field of Rabi frequency $R_m$ is considered perturbatively in the model to include the effect of MWMIC only. The weak probe field probes the coherence effects exhibited by the model when the strong driving fields are at exact resonance. The detuning parameter $\Delta _m$ associated with $R_m$ always assumes the same value as that of $\Delta _p$ due to the mutual relation of the detuning parameters of the fields involved in the model. That is why, for non-resonant cases, we have introduced the detuning for the probe only.

In presenting the results, we mainly concentrate on the absorption and dispersion of the weak probe field propagating through the atomic medium. In this connection, we plot the variation of real and imaginary parts of $\chi$ (as in Eq. (9)) in Fig. 2 against the probe detuning ($\Delta _p$) considering $R_n$ as a travelling-wave field. We present the variation of Im($\chi$) and Re($\chi$) in Fig. 2a when the field of Rabi frequency $R_m$ is absent between the two lowermost levels, while in Fig. 2b the said variation is shown under the application of $R_m$. The absence of the $R_m$ field leads the proposed system to a four-level DEIT model. The linear superposition of three states $|2>$, $|3>$, and $|4>$ by the two driving fields ($R_n$ and $R_c$) applied between the $|2>$-$|3>$ and $|3>$-$|4>$ transitions, respectively, results in three dressed states corresponding to the eigenvalues 0, $\pm \sqrt{R_c^2 + R_n^2}$. The occurrence of three prominent peaks at the detuning values $\Delta _p = 0, \pm 12.8$ in the absorption spectrum of Fig. 2a is attributed to the origination of these dressed states. The associated dispersion spectrum is shown by the dashed dot curve in Fig. 2a. We observe that a steep gradient region of the dispersion curve occurs around the line centre. When the $R_m$ is switched on, a close-loop configuration is formed and FWM-induced coherence effect is induced in the system. Absorption and dispersion profiles in such condition are presented in Fig. 2b. As is seen in the plot of Im($\chi$), the emergence of MWMIC in the system creates gain around the line centre leaving other side peaks of absorption with increased peak heights. This gain peak is the natural consequence of interplay between the non-linear term $f_{NL2}$ and the combination of the linear term $f_L$ and the other nonlinear term $f_{NL1}$. $f_{NL2}$ plays an opposite role in shaping the absorption spectrum with respect to $f_L$ and $f_{NL1}$. The dominance of the contribution of $f_{NL2}$ over the cumulative contribution of $f_L$ and $f_{NL1}$ leads to the generation of gain around the line centre. The absorption peaks at the detuning values $\Delta _p = \pm 12.8$ occur also as a result of different competitive effects. Actually the combination of $f_L$ and $f_{NL2}$ is responsible for the generation of these absorption peaks, though the presence of $f_{NL1}$ induces gain in the system. In this case, the dispersion spectrum is also modified, where the left wing of dispersion is dominated by the right wing around $\Delta _p$ = 0, which is unlike the case of Fig. 2a.

With a view to exhibiting the variation of diffraction intensity at the condition of multi-photon resonance *i*.*e*. $\Delta _p=\Delta _c=\Delta _n=0$, for $L=30$, Fig. 3 is presented. It shows the variation of intensity $I_\theta$ of different peaks in the grating structure in the normalised scale and the corresponding plots of the amplitude part |*T*(*x*)| and the phase part ${\Phi }(x)$ of $f({\xi },x)$. When the control field specified by the Rabi frequency $R_m$ is switched off, the curve of Fig. 3Ia depicts the pattern of grating-spectrum^51^ comprising of the complete evolution of nine peaks accompanied by the significantly intense central maximum and incomplete appearance of the marginal peaks (of the fifth order) near the both ends of sin($\theta$) axis. In this condition, the MWMIC-term does not contribute in shaping the grating-spectrum due to the absence of the spectral term $f_{NL2}$. In the presence of the nonlinear modulation term with $R_m=0.03$ MHz, the curve of Fig. 3Ib shows the appearance of almost equally enhanced peaks in the grating-spectrum. Such generation of the similar diffraction peaks in a uniformly distributed fashion occurs at the condition of exact atom-field resonance when the amplitude part of the grating-transfer-function $f({\xi },x)$ only contributes in the process while the phase part of $f({\xi },x)$ leaves no signature. This is remarkable to note that similar feature of diffraction pattern (Fig. 3II) mimics exclusively for the grating-transfer-function generated by the nonlinear modulation term $f_{NL2}$ for the same parametric condition of Fig. 3Ib. Physically, the occurrence of uniform distribution of peak heights implies that the distribution of photons over all the orders of diffraction peaks remains the same. To comprehend the role of $f({\xi },x)$, we have plotted Fig. 3III where the red-line curve denotes the variation in normalized amplitude transfer function (|*T*(*x*)|) and the dark-line signifies the null contribution of the phase function ${\Phi }(x)$.

On exploring such fascinating feature of uniform intensities for all the orders in the one-dimensional (1D) diffraction profile we have examined the diffraction pattern in two dimensions for the same set of values of parameters as chosen in Fig. 3Ib. The periodic arrangement of narrow diffracted light beams of nearly same intensity in the *x*-*y* plane is obtained (not shown). This unique spatial pattern of diffraction intensity can be envisioned as a 2D multi-channel beam splitter^33^ with potential applications in optical networking and communication, and also in all-optical device for multiplexing, demultiplexing^52^ etc. For the wide use of varied diffraction profiles in all-optical devices the detunings and the Rabi frequencies of the fields act as the control-knobs of the input-array pattern in meeting the varying needs of experimentalists for different purposes. Biased by the use of sub-diffraction limited spots in quantum lithography as employed in Ref.^53^, such 2D diffraction pattern may find special but limited application in high-precision optical lithography. Periodic pattern can be generated by the process of optical writing i.e., by exposing the desired surface of a substrate to the diffraction spots accompanied by periodic but same amount of etching of the illuminated zone of the surface.

To illustrate how the diffraction pattern evolves due to the resultant effect of two competitive components: amplitude and phase parts of $f({\xi },x)$ under the impact of nonlinear modulation, we plot Fig. 4 in near-resonant condition. In Fig. 4 the variation of intensity $I_\theta$ and the associated plots of the amplitude part |*T*(*x*)| and the phase part ${\Phi }(x)$ of $f({\xi },x)$ are presented in the left and right panels, respectively. When we switch on the knob of the nonlinear modulation i.e., fix $R_m$ at the same non-zero value (0.03 MHz) like Fig. 3, at the parametric condition ($\Delta _c=\Delta _n=0$, $\Delta _p=0.1$ MHz, and $L=30$) the grating-spectrum (curve a in the left panel) of Fig. 4I shows the monotonically decreasing nature of the diffraction-peak intensity from the extreme left end to the extreme right. This nature of intensity variation is reversed (curve *b* in the left panel of Fig. 4I) with the negative detuning $\Delta _p=-0.1$ MHz when the other parameters remain same. We note that the figure, as shown by the curves *a* and *b* together, exhibit evolution of gradually increasing / decreasing peak intensity from one end to another and thereby inducing asymmetry in peak pattern. For these two conditions, it is prominent in the right panel of Fig. 4I that the nature of the amplitude part |*T*(*x*)| does not change for the cases of the curves of *a* and *b*. However, the variation of phase part ${\Phi }(x)$ of the curve b is the mirror reflection of the curve a about a mirror plane placed parallel to the x axis at the zero position of the ${\Phi }(x)$-axis. Thus the mutually opposite variation in the intensity patterns of the curves (a and b of Fig. 4I) in the left panel indicates that such emergence of diffraction patterns are mainly due to the dominance of the phase part, ${\Phi }(x)$. When only the rate of detuning is increased to some higher value like $\Delta _p=4$ MHz for other fixed parameters, we obtain the enhanced peaks at the left half of sin$\theta$ axis as displayed by the curve *c* in left panel of Fig. 4II. The peaks in the right half are less significant in comparison with those in the left half. For more higher values of the detuning like $\Delta _p=12$ MHz, the feature of peak enhancement shows an opposite variation i.e., the peaks in the right half become prominent in comparison to the peaks arising in the left half as shown by the curve *d* in the left panel of Fig. 4II for the same values of the other parameters. The nature of variation of |*T*(*x*)| and ${\Phi }(x)$ in the right panel of Fig. 4II, does not predict the exact dominance of any of these two functions in the formation of such diffraction structure for each of the curves *c* and *d*.

In order to visualize the effect of the length of the active medium on the diffraction pattern at the detuned condition of the probe, we show the figures in the left and right panels of Fig. 5I,II,II both in the absence and presence of the nonlinear modulation terms, respectively, for the fixed values of the parameters like $\Delta _c=\Delta _n=0$. At the switched-off condition of the laser responsible for $R_m$ the curve shown in the left panel of Fig. 5I is dealt with the parameters: $\Delta _p=1.5$ MHz, and $L=60$. It is observed that the intensities of the peaks of non-zero order number get regularly enhanced with the increase in the order number, as we shift from the central peak of minimum intensity towards the fourth order diffraction peak. But the overall intensity of the peaks remains small. This is because of the predominant role of the phase part of $f({\xi },x)$ in the absence of $f_{NL2}$. In the presence of the control field with $R_m=0.02$ MHz, the curve given at the right panel of Fig. 5I gives rise to the evolution of asymmetric peaks with significantly reduced intensities of the peaks in the right half, which is due to the role of MWMIC in regulating the peak-pattern. With a small increase of the value of the probe detuning ($\Delta _p=3.5$ MHz) for the active medium with increasing length $L = 70$ in the absence of the $R_m$, the curve in the left panel of Fig. 5II shows that the central peak is almost suppressed with the occurrence of gradually enhanced higher order peaks on both sides of the grating spectrum. The inset shows the resulting diffraction pattern solely for the amplitude transfer function *T*(*x*) i.e. by setting the phase function $\Phi (x)$ zero. Only the central diffraction peak is prominently visible in the absence of phase modulation. This implies that the combined effect of amplitude and phase parts of the grating transfer function results in the diffraction pattern (left panel of Fig. 5II) with the suppression of central peak. The energy of the central component is being transferred into the higher order components of the diffraction pattern. The interplay between *T*(*x*) and $\Phi (x)$ is mainly responsible in reducing the intensity of the central peak and simultaneous enhancement of intensity of the higher order peaks. But, in the curve given at the right panel of Fig. 5II ($R_m=0.02$ MHz), we see that the nonlinear modulation of peak intensities by the MWMIC-effect increases the intensities of the peak-pattern as compared to that of Fig. 5I (right panel). For further increase in the detuning like $\Delta _p = 5.2$ MHz keeping $R_m$ field switched off and the other parameters same, it is observed that the curve exhibited in the left panel without MWMIC-effect becomes significantly modified in presence of MWMIC-effect as displayed by the curve in the right panel of Fig. 5III. This is to mention here that the modulation appearing in the peak intensities of the left half of the curve as shown in the right panel is significantly different when compared to that attained in the other two curves as shown in the right panel of Fig. 5I,II. Thus, we can infer that the term $f_{NL2}$ plays the key role to modify the intensity of the diffraction peak.

Based on the findings shown in Figs. 3, 4, 5 we intend to summarize the key features related of the diffraction spectra as a whole. In Fig. 3Ia the diffraction profile shows symmetric distribution of peaks around the central ($sin(\theta )$ = 0) peak with varying peak heights at exact resonance of all the fields. But the application of the field of Rabi frequency $R_m$ induces MWMIC in the closed system and thereby leads to the generation of uniformly distributed multiple peaks of nearly same intensity (Fig. 3Ib. It is the most interesting outcome of the present study obtained at multi-photon resonance condition. Setting the probe at near resonance, it is also surprising to note that the profile of peaks with almost same diffraction intensity loses symmetry and evolves into an asymmetric spectrum with diffraction intensities increasing or decreasing gradually (Fig. 4a,b). If the laser responsible for $R_m$ is turned on and off, remarkable changes are found when both the detuning parameter of the probe field and the sample length are changed simultaneously. We obtain an interesting feature like the suppression of central diffraction peak in symmetric alignment of multiple peaks in the absence of $R_m$ (left panel of Fig. 5II), when the probe is non-resonant. We infer that very less number of probe photons responsible for the zeroth order peak get diffracted on spreading into the higher order peaks. When the FWM-induced coherence effect is present in the system due to $R_m$, symmetry in diffraction profiles is destroyed due to non-resonant probe, as is prominent in the right panel of Fig. 5.

**Figure 6:**
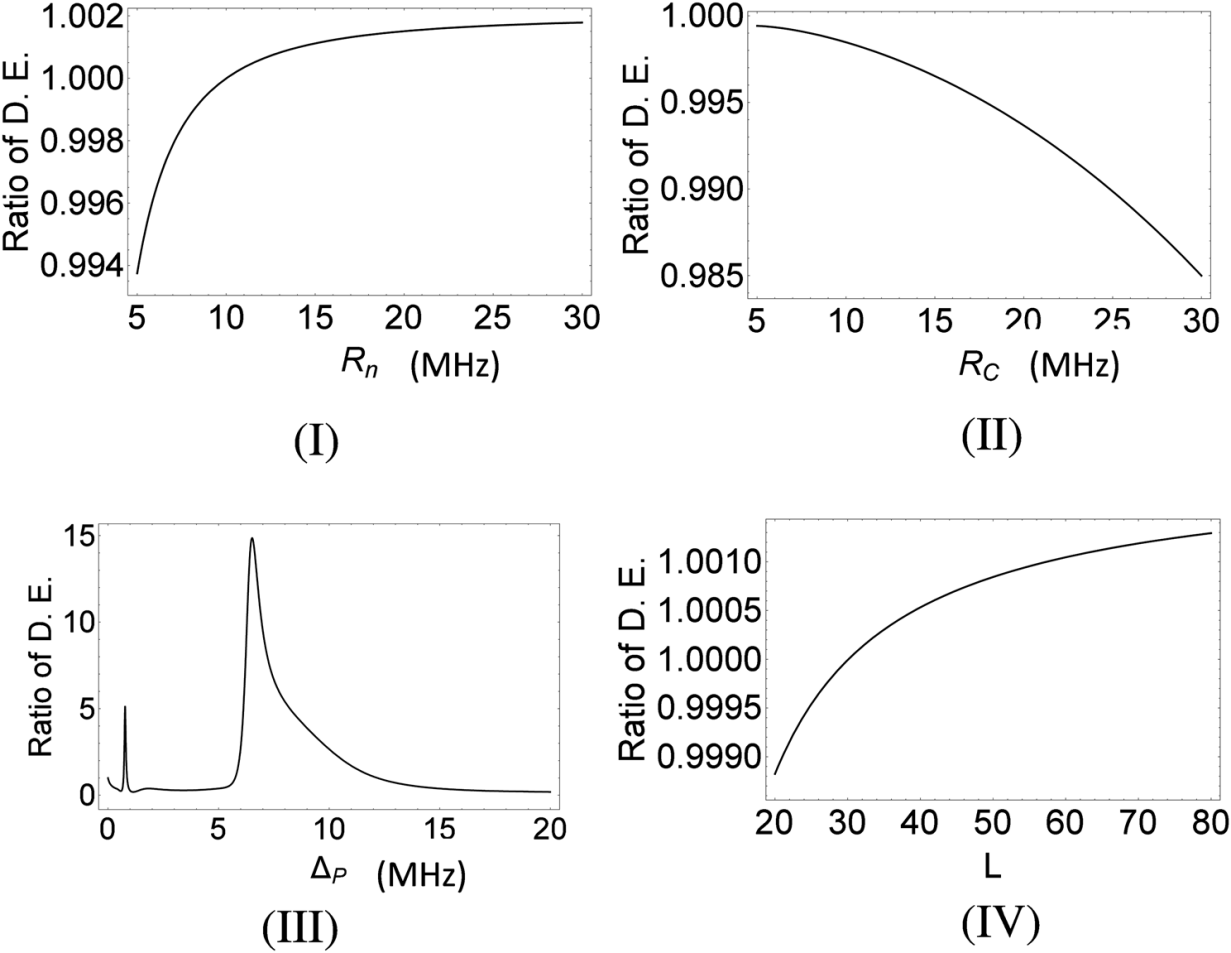
Ratio of diffraction efficiencies (D.E.) to show the dependence of Rabi frequencies ($R_n$ and $R_c$), probe-detuning ($\Delta _p$) and sample length (dimensionless, see text) for $i = 4$ for the fourth order peak at sin$(\theta )$ = 0.8 and $j = 1$ for the first order peak at sin$(\theta )$ = 0.2 in Fig. 3Ib. All the parameters are same as in Fig. 3Ib except the variable used.

It is well known that, for the evolution of *i*-th peak in the grating spectrum, $I(\theta _i)$/$I_0$ measures the diffraction efficiency (D.E.)^8^ for that particular diffraction peak. In this context, we have drawn the ratio of DE *i*.*e*. $I(\theta _i)$/ $I(\theta _j)$ ($i>j$)in Fig. 6 for *i*=4(sin$(\theta )$ = 0.8) and *j*=1(sin$(\theta )$ = 0.2). Figure 6I,II,III,IV exhibit the mentioned ratio of D.E. with the increase of the values of the controlling parameters like the Rabi frequencies: $R_n$ (Fig. 6I), $R_c$ (Fig. 6II), the detuning parameter $\Delta _p=\Delta$ (Fig. 6III) and the length *L* of the active medium (Fig. 6IV) for fixed values of the parameters like $\Delta _c=\Delta _n=0$, $R_p=0.01$ MHz and $R_m=0.03$ MHz. The value of *L* is set as 30 for Fig. 6I–III. As depicted by Fig. 6I ($\Delta =0$, $R_c=8$ MHz), the intensity ratio rapidly increases up to $R_n$ $=$ 10 MHz and then both the diffraction intensities $I(\theta _1)$ and $I(\theta _4)$ become nearly equal for larger values of $R_n$. If we plot the intensity ratio versus the Rabi frequency $R_c$, we obtain the curve as presented by Fig. 6II ($\Delta =0$, $R_n=5$ MHz). It is observed that with the increase in the value of $R_c$, $I(\theta _4)$ slowly decreases as compared to the variation in $I(\theta _1)$. A double-peak like structure is obtained in the variation of the intensity ratio with the probe-detuning as depicted in the plot of Fig. 6III with $R_n=10$ MHz and $R_c=8$ MHz. This implies that higher order peak intensity is significantly enhanced with respect to that of the first order near the peaks at around the probe detuning of 1 MHz and 6 MHz. In between the two peaks the ratio is very small. This feature can be attributed to the negative impact of the interplay between the amplitude and phase parts of the transfer function $f({\xi },x)$ on the formation of the diffraction pattern. In Fig. 6IV ($\Delta =5.2$ MHz, $R_n=10$ MHz and $R_c=8$ MHz), we have shown the interesting feature for the variation of the intensity ratio with the increase in the length of the active medium. This implies that the first- and fourth- order diffraction intensities slowly evolve to the same value with the increase in sample length. Thus, it can be concluded that proper choice of the values of the controlling parameters involved in the system leads us to obtain sufficiently intense higher order diffraction peak intensities at resonant as well as off-resonant conditions of the probe field.

The basic mechanism leading to EIG in the present scheme is based on multi-wave-mixing-induced coherence effect. So far we have discussed all the results in the life-time broadened regime. In the presence of Doppler Broadening, the appearance of phase dephasing collision rates becomes prominent in all the atomic transitions considered in the atomic model, which significantly affects the atomic coherence needed for obtaining the symmetric diffraction pattern. The incorporation of considerable Doppler width ($\sim$ a few MHz) results in a significant decrease in the intensity of diffraction peaks in the grating pattern as a natural consequence of suppression of nonlinear wave-mixing-induced coherence effect. Experiments conducted in cold atoms on the basis of the present model is expected to realize EIG with sufficiently improved efficiency.

## Conclusion

We have studied nonlinear modulation effect on the diffraction pattern of EIG as a result of spatially modulated four-wave-mixing induced coherence effect in a four-level close-loop interaction system. In order to enhance the applicability of the proposed scheme in practice, Rubidium 87$D_1$ transitions are taken into account to obtain the grating pattern. We note that the amplitude part of the grating-transfer-function plays the vital role in forming significantly enhanced diffraction peaks of nearly same height at the condition of exact atom-field resonance. It has been described how peak-asymmetry results in the system at the detuned condition of the probe field. This is interesting to observe that higher order peaks can be much more enhanced than that of the lower order peaks due to nonlinear peak modulation effect. We have shown that, with the variation of the length of the active medium, the diffraction pattern obtained at a particular parametric condition can be changed abruptly. The method of achieving controllable peak-pattern may be an useful technique for generation, control and detection of nonlinear light originated by the spatially modulated four-wave-mixing process. The variation in peak-pattern suggests that the present model may be applied in the cases of all-optical devices, optical networking, and for a specified purpose in high-precision atom lithography.

## References

[1] Harada KI, Tanaka S, Kanbashi T, Mitsunaga M, Motomura K (2005). Electromagnetically induced diffraction in sodium vapor. Opt. Lett..

[2] Mitsunaga M, Imoto N (1999). Observation of an electromagnetically induced grating in cold sodium atoms. Phys. Rev. A.

[3] Cardoso GC, Tabosa JWR (2002). Electromagnetically induced gratings in a degenerate open two-level system. Phys. Rev. A.

[4] Ling HY, Li YQ, Xiao M (1998). Electromagnetically induced grating: Homogeneously broadened medium. Phys. Rev. A.

[5] Sheng J, Wang J, Mairi M-A, Christodoulides DN, Xiao M (2015). Observation of discrete diffraction patterns in an optically induced lattice. Opt. Exp..

[6] Wen F, Ye H, Zhang X, WANG W, Li S, Wang H, Zhang Y, Qiu C.-W (2017). Optically induced atomic lattice with tunable near-field and far-field diffraction patterns. Photon. Res..

[7] Xiao Z-H, Shin SG, Kim K (2010). An electromagnetically induced grating by microwave modulation. J. Phys. B At Mol. Opt. Phys..

[8] de Araujo LEE (2010). Electromagnetically induced phase grating. Opt. Lett..

[9] Marangos JP (1998). Electromagnetically induced transparency. J. Mod. Opt..

[10] Lukin MD, Yelin SF, Fleischhauer M, Scully MO (1999). Quantum interference effects induced by interacting dark resonances. Phys. Rev. A.

[11] Mulchan N, Ducreay DG, Pina R, Yan M, Zhu Y (2000). Nonlinear excitation by quantum interference in a Doppler-broadened rubidium atomic system. J. Opt. Soc. Am. B.

[12] Ficek Z, Swain S (2005). Quantum interference and coherence (Springer series in optical sciences).

[13] Brown AW, Xiao M (2005). All-optical switching and routing based on an electromagnetically induced absorption grating. Opt. Lett..

[14] Dutta BK, Mahapatra PK (2006). Electromagnetically induced grating in a three-level -type system driven by a strong standing wave pump and weak probe fields. J. Phys B: At. Mol. Opt. Phys..

[15] Wan, R. G., Kou, J., Jiang, L., & Gao J. Y. Electromagnetically induced grating via enhanced nonlinear modulation by spontaneously generated coherence. *Phys. Rev. A* **83033824**, (2011).

[16] Naseri T, Bonabi SR (2014). Efficient electromagnetically induced phase grating via quantum interference in a four-level N-type atomic system. J. Opt. Soc. Am.B.

[17] Vafafard A, Mahmoudi M (2015). Switching from electromagnetically induced absorption grating to electromagnetically induced phase grating in a closed-loop atomic system. Appl. Opt..

[18] Bonabi SR, Naseri T (2015). Theoretical investigation of electromagnetically induced phase grating in RF-driven cascade-type atomic systems. Appl. Opt..

[19] Bozorgzadeh F, Saharai M, Khoshsima H (2016). Controlling the electromagnetically induced grating via spontaneously generated coherence. Eur. Phys. J. D.

[20] Wang L, Qi Y-H, Deng L, Niu Y-P, Gong S-Q, Guo H-J (2017). Effect of Phase Modulation on Electromagnetically Induced Grating in a Five-Level M-Type Atomic System. Chin. Phys. Lett..

[21] Chen Y-Y, Liu Z-Z, Wan R-G (2017). Electromagnetically Induced Grating Without Absorption Using Incoherent Pump. Int. J Theor. Phys..

[22] Zhao L (2018). Electromagnetically induced polarization grating. Sci. Rep..

[23] Asghar S, Ziauddin Qamar S, Qamar S (2016). Electromagnetically induced grating with Rydberg atoms. Phys. Rev. A.

[24] Ma D, Yu D, Zhao X.-D, Qian J (2019). Unidirectional and controllable higher-order diffraction by a Rydberg electromagnetically induced grating.. Phys. Rev. A.

[25] Cheng G-L, Zhong W-X, Chen A-X (2015). Phonon induced phase grating in quantum dot system. Opt. Exp..

[26] Zhao L, Duan W, Yelin SF (2010). All-optical beam control with high speed using image-induced blazed gratings in coherent media. Phys. Rev. A.

[27] Zhang Z, Li F, Malpuech G, Zhang Y, Bleu O, Koniakhin S, Li C, Zhang Y, Xiao M, Solnyshkov DD (2019). Particlelike Behavior of Topological Defects in Linear Wave Packets in Photonic Graphene. Phys. Rev. Lett..

[28] Zhang Z, Liang S, Li F, Ning S, Li Y, Malpuech G, Zhang Y, Xiao M, Solnyshkov D (2020). Spin-orbit coupling in photonic grapheme. Optica.

[29] Zhang Z, Wang R, Zhang Y, Kartashov YV, Li F, Zhong H, Guan Gao K, Li F, Zhang Y, Xiao M (2020). Observation of edge solitons in photonic grapheme. Nat. Comm..

[30] Shui T, Yang W-X, Liu S, Ling L, Zhu Z (2018). Asymmetric diffraction by atomic gratings with optical $${\cal{PT}}$$ symmetry in the Raman-Nath regime. Phys. Rev. A.

[31] Liu Y-M, Gao F, Fan C-H, Wu J-H (2017). Asymmetric light diffraction of an atomic grating with $${\cal{PT}}$$ symmetry. Opt. Lett..

[32] Wang L, Zhou F, Hu P, Niu Y, Gong S (2014). Two-dimensional electromagnetically induced cross-grating in a four-level tripod-type atomic system. J. Phys. B: At. Mol. Opt. Phys..

[33] Chen Y, Liu Z, Wan R (2016). Two-dimensional electromagnetically induced grating in coherent atomic medium. Eur. Phys. Lett..

[34] Vafafard A, Sahrai M (2018). Electromagnetically induced grating based on Zeeman coherence oscillations in cases beyond the multi-photon resonance condition. J. Opt. Soc. Am. B.

[35] Yuan J, Wu C, Wang L, Chen G, Jia S (2019). Observation of diffraction pattern in two-dimensional optically induced atomic lattice. Opt. Lett..

[36] Wen J, Du S, Chen H, Xiao M (2011). Electromagnetically induced Talbot effect. Appl. Phys. Lett..

[37] Zhang Z, Liu X, Zhang D, Sheng J, Zhang Y, Zhang Y, Xiao M (2018). Observation of electromagnetically induced Talbot effect in an atomic system. Phys. Rev. A.

[38] Zhou F, Qi Y, Sun H, Chen D, Yang J, Niu Y, Gong S (2013). Electromagnetically induced grating in asymmetric quantum wells via Fano interference. Opt. Express.

[39] Naseri T (2016). Two-dimensional induced grating in Rydberg atoms via microwave field. Superlatt. Microstruct..

[40] Naseri T (2017). Investigation of dual electromagnetically induced grating based on spatial modulation in quantum well nanostructures via biexciton coherence. Laser Phys..

[41] Bozorgzadeh F, Sahrai M (2019). Laser-induced diffraction grating in asymmetric double quantum well nanostructure. Laser Phys. Lett..

[42] Naseri T (2020). Electromagnetically induced grating in semiconductor quantum dot and metal nanoparticle hybrid system by considering nonlocality effects. J. Theo. Appl. Phys..

[43] Naseri T (2020). Optical properties and electromagnetically induced grating in a hybrid semiconductor quantum dot-metallic nanorod system. Phys. Lett. A.

[44] Al-Salihi FR, Al-Khursan AH (2020). Electromagnetically induced grating in double quantum dot system. Opt. Quan. Elec..

[45] Vafafard A, Sahrai M (2020). Tunable double electromagnetically induced grating with an incoherent pump field. J. Opt. Soc. Am. B.

[46] Steck, D. A. Rubidium 87 D line data, available online at [http://steck.us/alkalidata]

[47] Dowling JP, Bowden CM (1993). Near dipole-dipole effects in lasing without inversion: An enhancement of gain and absorptionless index of refraction. Phys. Rev. Lett..

[48] Steward EG (2004). Fourier Optics: An Introduction.

[49] In ref. [14], the Expression (17) should contain the term $$|F(\theta )|^2$$ instead of $$F(\theta )$$. This is a typographical mistake.

[50] Tian S-C, Wan R-G, Wang L-J, Shu S-L, Lu H-Y, Zhang X, Tong C-Z, Feng J-L, Xiao M, Wang L-J (2018). Asymmetric light diffraction of two-dimensional electromagnetically induced grating with PT symmetry in asymmetric double quantum wells. Opt. Express.

[51] The word ‘spectrum’ is generally used for the distribution of intensities over wavelengths. Here, ‘grating-spectrum’ implies the appearance of the angular distribution of diffraction peaks over the variation of spatial phase at the output of the grating.

[52] Yang J, Jiang X, Wang M, Wang Y (2004). Two-dimensional wavelength demultiplexing employing multilevel arrayed waveguides. Opt. Express.

[53] Peer A, Dayan B, Vucelja M, Silberberg Y, Friesem AA (2004). Quantum lithography by coherent control of classical light pulses. Opt. Express.

