# Author Correction: Multi-wave-mixing-induced nonlinear modulation of diffraction peaks in an opto-atomic grating

**DOI:** 10.1038/s41598-024-55638-w

**Published:** 2024-03-06

**Authors:** Bibhas Kumar Dutta, Pradipta Panchadhyaayee, Indranil Bayal, Prasanta Kumar Mahapatra, Nityananda Das

**Affiliations:** 1 Department of Physics, Sree Chaitanya College (WB State University), North 24 Parganas, Habra, WB 743 268 India; 2 Department of Physics (UG & PG), Prabhat Kumar College (Vidyasagar University), Contai, Purba Medinipur, 721404 India; 3 ITER, Siksha ‘O’ Anusandhan University, Bhubaneswar, Odisha 751030 India; 4 Department of Physics, J. K. College (Sidho Kanho Birsha University), Purulia, WB 723 101 India

Correction to: *Scientific Reports* 10.1038/s41598-020-73825-3, published online 08 October 2020

The original version of this Article contains an error in clarification of the constant, *C*. As a result of this error, in the Theoretical model section,

“Now, the Maxwell’s equation for the probe field is given in the following form^12^

10 $$\frac{\delta {\epsilon }_{p}}{\delta z}=i\frac{\pi }{{\epsilon }_{0}{\lambda }_{p}}{P}_{p}=iC\frac{\chi }{{\lambda }_{p}}{\epsilon }_{p}$$

where $C= \frac{\pi N{\left|{\mu }_{14}\right|}^{2}}{{\epsilon }_{0}\hbar {\gamma }_{41}}$ is defined as a dimensionless constant by following the Ref.^12,47^, which implies the optical depth of the probe field.”

should read:

“Now, the Maxwell’s equation for the probe field is given in the following form^12^

10 $$\frac{\delta {\epsilon }_{p}}{\delta z}=i\frac{\pi }{{\epsilon }_{0}{\lambda }_{p}}{P}_{p}=iC\frac{\chi }{{\lambda }_{p}}{\epsilon }_{p}$$

where $C= \frac{\pi N{\left|{\mu }_{14}\right|}^{2} }{{\epsilon }_{0}\hbar {\gamma }_{41}}$ is defined as a dimensionless constant by following the Ref.^12,47^.”

“We introduce the dimensionless distance (*ξ*) and relate it with the optical depth of probe field (*C*) through the expression, $\upxi =\frac{zC}{{\lambda }_{p}}$.”

should read:

“We introduce the dimensionless distance (*ξ*) and relate it with the dimensionless constant (*C*) through the expression, $\upxi =\frac{zC}{{\lambda }_{p}}$.”

